# The Absence of Intra‐Tumoral Tertiary Lymphoid Structures is Associated with a Worse Prognosis and mTOR Signaling Activation in Hepatocellular Carcinoma with Liver Transplantation: A Multicenter Retrospective Study

**DOI:** 10.1002/advs.202309348

**Published:** 2024-03-18

**Authors:** Jianhua Li, Li Zhang, Hao Xing, Yan Geng, Shaocheng Lv, Xiao Luo, Weiqiao He, Zhi Fu, Guangming Li, Bin Hu, Shengran Jiang, Zhe Yang, Ningqi Zhu, Quanbao Zhang, Jing Zhao, Yifeng Tao, Conghuan Shen, Ruidong Li, Feng Tang, Shusen Zheng, Yun Bao, Qiang He, Daoying Geng, Zhengxin Wang

**Affiliations:** ^1^ Liver Transplantation Center Department of General Surgery Huashan Hospital Fudan University Shanghai 200040 P. R. China; ^2^ Institute of Organ Transplantation Fudan University Shanghai 200040 P. R. China; ^3^ Shanghai Institute of Immunology Department of Immunology and Microbiology Shanghai Jiao Tong University School of Medicine Shanghai 200025 P. R. China; ^4^ Hepatobiliary Surgery Department of General Surgery Huashan Hospital & Cancer Metastasis Institute Fudan University Shanghai 200040 P. R. China; ^5^ Department of Hepatobiliary Surgery Beijing Chaoyang Hospital affiliated to Capital Medical University Beijing 100020 P. R. China; ^6^ Academy for Engineering and Technology Fudan University Shanghai 200032 P. R. China; ^7^ General Surgery Center Beijing Youan Hospital Capital Medical University Beijing 100020 P. R. China; ^8^ Department of Radiology Huashan Hospital Fudan University Shanghai 200040 P. R. China; ^9^ Institute of Functional and Molecular Medical Imaging Fudan University Shanghai 200040 P. R. China; ^10^ Department of Hepatobiliary and Pancreatic Surgery, Shulan (Hangzhou) Hospital Zhejiang Shuren University School of Medicine Hangzhou 310022 P. R. China; ^11^ Department of Critical Care Medicine Huashan Hospital Fudan University Shanghai 200040 P. R. China; ^12^ Department of Pathology, Huashan Hospital Fudan University Shanghai 200040 P. R. China

**Keywords:** hepatocellular carcinoma, liver transplantation, mTOR signaling, radiomics, tertiary lymphoid structure

## Abstract

Tertiary lymphoid structure (TLS) can predict the prognosis and sensitivity of tumors to immune checkpoint inhibitors (ICIs) therapy, whether it can be noninvasively predicted by radiomics in hepatocellular carcinoma with liver transplantation (HCC‐LT) has not been explored. In this study, it is found that intra‐tumoral TLS abundance is significantly correlated with recurrence‐free survival (RFS) and overall survival (OS). Tumor tissues with TLS are characterized by inflammatory signatures and high infiltration of antitumor immune cells, while those without TLS exhibit uncontrolled cell cycle progression and activated mTOR signaling by bulk and single‐cell RNA‐seq analyses. The regulators involved in mTOR signaling (RHEB and LAMTOR4) and S‐phase (RFC2, PSMC2, and ORC5) are highly expressed in HCC with low TLS. In addition, the largest cohort of HCC patients is studied with available radiomics data, and a classifier is built to detect the presence of TLS in a non‐invasive manner. The classifier demonstrates remarkable performance in predicting intra‐tumoral TLS abundance in both training and test sets, achieving areas under receiver operating characteristic curve (AUCs) of 92.9% and 90.2% respectively. In summary, the absence of intra‐tumoral TLS abundance is associated with mTOR signaling activation and uncontrolled cell cycle progression in tumor cells, indicating unfavorable prognosis in HCC‐LT.

## Graphical abstract

This study by Jianhua Li, Li Zhang, Hao Xing, and co‐workers reveals that the absence of intra‐tumoral tertiary lymphoid structure (TLS) is associated with enhanced mTOR signaling activation, indicating an unfavorable prognosis in hepatocellular carcinoma with liver transplantation (HCC‐LT). A radiomics‐based classifier capable of noninvasively and accurately distinguishing high TLS samples and low ones is built, serving as a biomarker for evaluating prognosis and assessing responses to pre‐transplant treatments.

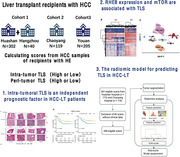

## 1 Introduction

Hepatocellular carcinoma (HCC), the most common form of primary liver cancer, is the leading cause of cancer‐related deaths.^[^ ^1^ ^]^ Approximately fifty percent of such fatalities transpire in the realm of China, wherein 80% of afflicted individuals bear the burden of HBV infection, with an overwhelming 85% of the demographic being of the male persuasion.^[^ ^2^, ^3^ ^]^

Liver transplantation emerges as the curative therapy for HCC with cirrhosis, effecting a dual remedy by eradicating both tumors and the conducive microenvironment fostering carcinogenesis. Nevertheless, the pronounced prevalence of recurrence and metastasis following liver transplantation for HCC (HCC‐LT) has impeded the attainment of sustained long‐term survival. The median overall survival period stands at 13 months following the initiation of tumor recurrence and metastasis, underscoring the critical imperative to accurately discern high‐risk cohorts susceptible to such metastatic resurgence.^[^ ^4^ ^]^

Despite the numerous criteria proposed for predicting prognosis and selecting beneficiaries for HCC‐LT, there remains a shortage of biomarkers capable of accurately predicting outcomes and responses to pre‐transplant treatments. This gap is particularly evident in patients beyond Milan criteria, where tumor burden is insufficient to predict recurrence‐free survival, and yet pre‐transplant therapy is necessary defining the population that would benefit from such therapy proves challenging. Other factors such as tumor heterogeneity, circulating tumor cells and tumor‐infiltrating immune cells play a significant role, but the effectiveness lacks validation in multicenter, large cohort studies of HCC‐LT.^[^ ^5^, ^6^ ^]^ Moreover, the effective way to predict it is still unclear, incorporating biological and morphological markers of response to pre‐transplant treatments during the waiting period, especially considering that systemic therapies like immune checkpoint inhibitors (ICIs) treatment is increasingly used for downstaging or bridge therapy before HCC‐LT.

In recent years, TLS, defined as the group of immune cell aggregates formed in non‐lymphoid tissues, has attracted particular interest, as classification based on TLS states could efficiently stratify patients into subgroups with distinct clinical outcomes in multiple malignancies^[^ ^7^, ^8^, ^9^ ^]^ In HCC, the presence of either intra‐tumoral TLS or peri‐tumoral TLS is found to be associated with improved survival and enhanced immune responses,^[^ ^10^ ^]^ giving us a hint that TLS may also be a promising biomarker for the prediction of prognosis and drug response in HCC‐LT. It is well recognized that the TLS plays an impressive part in the adaptive antitumor immune response as they resemble secondary lymphoid organs (SLOs) in both anatomical and functional aspects.^[^ ^11^ ^]^ However, the molecular characteristics of cancer cells and the tumor microenvironment (TME) in HCC without TLS have not been fully clarified.

Given that TLS is a promising biomarker for predicting cancer prognosis and drug response, accurate detection is essential in clinical practice. The histopathological measurement is a golden standard for TLS detection; however, biopsies could trigger adverse events such as pain, bleeding, tumor metastasis, and, on infrequent occasions, mortality.^[^ ^12^ ^]^ By contrast, radiomic is expected to be an auxiliary tool to assist diagnosis and surveillance in a non‐invasive manner. A previous study has demonstrated the predictive potentials of a CT‐based nomogram in predicting the presence of intra‐tumoral TLS in HCC,^[^ ^13^ ^]^ suggesting a possibility to infer TLS states through image‐derived morphometrics.

To assess the prognostic implications of TLS in the context of hepatocellular carcinoma after liver transplantation (HCC‐LT), we conducted a retrospective compilation of clinical data, digital representations of tumors of hematoxylin and eosin (HE) staining for HCC‐LT patients across four distinguished medical centers. The objective was to elucidate the correlation between intra‐tumoral and peri‐tumoral TLS abundance and the prognostic trajectory of HCC‐LT. Additionally, we quantified intra‐tumoral TLS prevalence within the Cancer Genome Atlas (TCGA) cohort using digital pathology HE staining and probed the molecular attributes of HCC devoid of TLS manifestation.

Our findings were subsequently corroborated through comprehensive validation in single‐cell sequence datasets and matched tissue samples derived from the Huashan cohort. Leveraging radiomic CT data, we constructed a radiomic model of considerable efficacy in predicting TLS abundance. This non‐invasive approach, circumventing the necessity for liver biopsies, amalgamates histological observations garnered through imaging modalities with molecular analyses. It holds promise in furnishing precise prognostic classifications and therapeutic stratagems for individuals undergoing HCC‐LT, thereby advancing the landscape of personalized therapeutic interventions.

## 2 Results

### 2.1 Baseline Characteristics of Patients in Three HCC Cohorts

A total of 666 patients (302 patients from Huashan cohort, 119 from Chaoyang cohort, 40 from Hangzhou cohort, and 205 from Youan cohort) who underwent HCC‐LT were enrolled in this study. Due to the regional features of eastern China and relatively restrictive sample size, the Huashan and Hangzhou cohorts were merged into one cohort, namely the HSHZ cohort. Overall, 602 (90.4%) patients were men, and 144 (21.6%) patients were over 60 years old. The median follow‐up time was 27.9, 25, and 29.5 months for cohorts Huashan and Hangzhou (HSHZ), Chaoyang, and Youan, respectively. Among those demographic characteristics, blood type, sex, alpha‐fetoprotein (AFP) levels, hepatitis B and hepatitis C virus infection, total bilirubin (Tbil) are not different among the centers (**Table** **1**). However, the proportions of patients beyond Milan and UCSF criteria, with microvascular invasion (MVI) presence, with portal vein tumor thrombus (PVTT), with tumor diameter over 5 centimeters were higher in the HSHZ cohort.

**Table 1:** The demographic and clinical characteristics of HSHZ, Chaoyang and Youan HCC‐LT cohorts.

Variables	HSHZ (*n* = 342)	Chaoyang (*n* = 119)	Youan (*n* = 205)	*P value*
Blood type				
A	78 (26%)	27 (22.7%)	57 (27.8%)	1.97E‐01
AB	27 (9%)	17 (14.3%)	32 (15.6%)	
B	93 (31%)	40 (33.6%)	63 (30.7%)	
O	102 (34%)	35 (29.4%)	53 (25.9%)	
Gender				
Female	30 (8.8%)	12 (10.1%)	22 (10.7%)	7.39E‐01
Male	312 (91.2%)	107 (89.9%)	183 (89.3%)	
Age				
≤60	269 (82.8%)	80 (67.2%)	156 (76.1%)	**1.75E‐03**
>60	56 (17.2%)	39 (32.8%)	49 (23.9%)	
AFP				
≤200	201 (67.2%)	88 (73.9%)	138 (67.3%)	3.68E‐01
>200	98 (32.8%)	31 (26.1%)	67 (32.7%)	
PT				
≤13	138 (41.7%)	33 (27.7%)	80 (39%)	**2.63E‐02**
>13	193 (58.3%)	86 (72.3%)	125 (61%)	
HBsAg				
Negative	62 (20.5%)	43 (36.1%)	45 (22%)	**2.38E‐03**
Positive	240 (79.5%)	76 (63.9%)	160 (78%)	
HCV				
Negative	294 (97.4%)	115 (96.6%)	192 (93.7%)	1.06E‐01
Positive	8 (2.6%)	4 (3.4%)	13 (6.3%)	
Tbil				
≤23	159 (49.8%)	47 (39.5%)	87 (42.4%)	8.51E‐02
>23	160 (50.2%)	72 (60.5%)	118 (57.6%)	
Alb				
≤35	93 (29.2%)	62 (52.1%)	86 (42%)	**1.64E‐05**
>35	226 (70.8%)	57 (47.9%)	119 (58%)	
PLT				
≤100	157 (49.5%)	74 (62.2%)	36 (17.6%)	**1.35E‐17**
>100	160 (50.5%)	45 (37.8%)	169 (82.4%)	
AJCC				
T1‐T2	233 (68.1%)	104 (87.4%)	163 (79.5%)	**3.32E‐05**
T3‐T4	109 (31.9%)	15 (12.6%)	42 (20.5%)	
Milan criteria				
No	236 (69%)	56 (47.1%)	115 (56.1%)	**2.72E‐05**
Yes	106 (31%)	63 (52.9%)	90 (43.9%)	
UCSF criteria				
No	187 (54.7%)	27 (22.7%)	99 (48.3%)	**1.21E‐08**
Yes	155 (45.3%)	92 (77.3%)	106 (51.7%)	
Tumor Number				
>1	168 (49.1%)	44 (37%)	123 (60%)	**2.81E‐04**
1	174 (50.9%)	75 (63%)	82 (40%)	
Max tumor diameter (cm)				
≤5	216 (63.2%)	91 (76.5%)	143 (69.8%)	**2.04E‐02**
>5	126 (36.8%)	28 (23.5%)	62 (30.2%)	
MVI				
No	89 (26%)	50 (42%)	145 (70.7%)	**1.78E‐23**
Yes	253 (74%)	69 (58%)	60 (29.3%)	
PVTT				
No	257 (75.1%)	107 (89.9%)	183 (89.3%)	**8.29E‐06**
Yes	85 (24.9%)	12 (10.1%)	22 (10.7%)	
Differentiation				
G1‐G2	177 (63.9%)	92 (78.6%)	105 (61%)	**4.62E‐03**
G3‐G4	100 (36.1%)	25 (21.4%)	67 (39%)	
Intra‐tumoral TLS				
High	170 (49.7%)	60 (50.4%)	79 (38.5%)	**2.50E‐02**
Low	172 (50.3%)	59 (49.6%)	126 (61.5%)	
Peri‐tumoral TLS				
High	220 (64.3%)	106 (89.1%)	150 (73.2%)	**1.41E‐06**
Low	122 (35.7%)	13 (10.9%)	55 (26.8%)	

AFP, α‐fetoprotein; PT, Prothrombin time; PLT, Platelet; AJCC, American Joint Committee on Cancer; UCSF, University of California San Francisco; MVI, microvascular invasion; PVTT, Portal vein tumor thrombus.

### 2.2 Intra‐Tumoral TLS Abundance has a Higher Prognostic Value than Its Peri‐Tumoral Abundance

**Figure 1:**
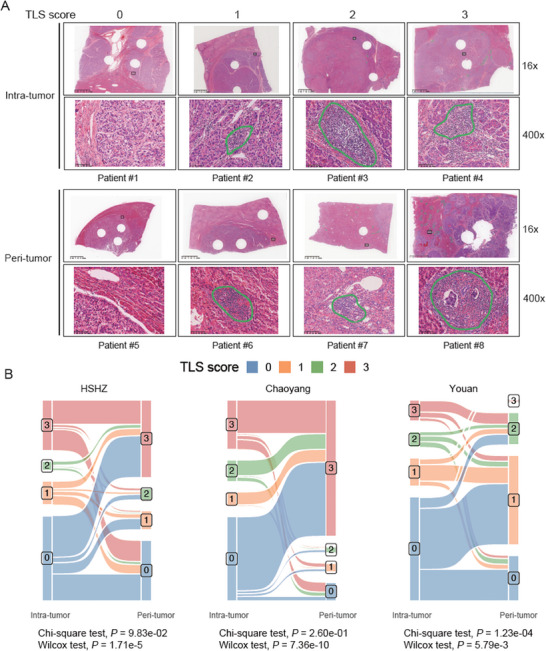
The tertiary lymphoid structure (TLS) scoring system. A) Representative whole slide images of different TLS abundances (TLS scores from 0 to 3). B) Significant associations were found between the intra‐tumoral and peri‐tumoral TLS scores for cohorts HSHZ (*n* = 342) and Chaoyang (*n* = 119) and Youan (*n* = 205), (*P* < 0.05). The *p*‐values were calculated by chi‐square test.

The TLS abundance in intra‐tumoral and peri‐tumoral regions was rated according to our scoring system (**Figure** **1**A, See Experimental Section). The intra‐tumoral TLS abundance was significantly reduced compared to the peri‐tumoral TLS abundance in all three cohorts (Figure 1B, Wilcoxon test, *P* < 0.05). Furthermore, the TLS abundance across intra‐tumoral and peri‐tumoral regions exhibited little consistency in HSHZ and Chaoyang HCC‐LT cohorts (Figure 1B, Chi‐square test, *P* > 0.05). In contrast, it showed consistency in Youan cohort (Figure 1B, Chi‐square test, *P* < 0.05). To investigate why there was a difference in the consistency of TLS abundance between intra‐tumoral and peri‐tumoral regions among the three cohorts, we divided the HCC samples into three groups based on the extent of decrease in TLS between intra‐tumoral and peri‐tumoral regions: significantly decreased (SD, difference < −1), not significantly changed (NS, difference between −1 and 1), and significantly increased (SI, difference >1). It is evident that the proportion of SD individuals in the Youan cohort was the lowest (Figure S1A, Supporting Information). Additionally, SD group had a higher proportion of MVI‐positive patients (Figure S1B, Supporting Information), while the Youan cohort had the lowest proportion of MVI‐positive cases (Figure S1C, Supporting Information). Therefore, we speculated that the higher consistency of the TLS abundance across intra‐tumoral and peri‐tumoral regions in Youan cohort might be associated with a lower proportion of MVI positivity rate.

**Figure 2:**
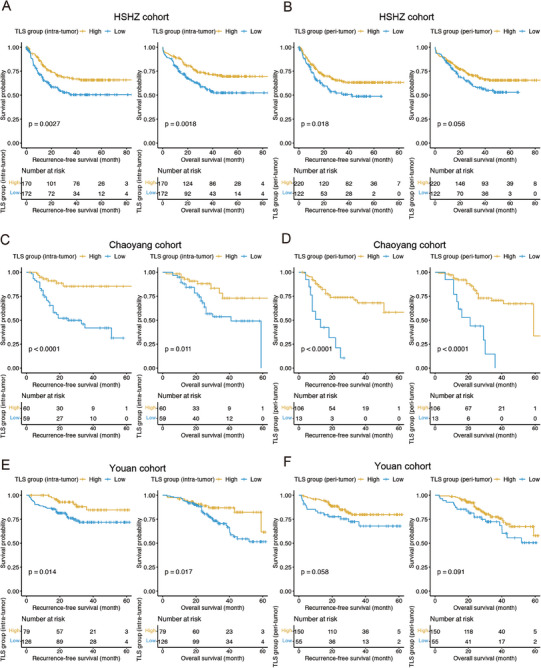
Kaplan‐Meier estimates of recurrence‐free survival and overall survival according to intra‐tumoral and peri‐tumoral TLS abundance. A, B) The Kaplan–Meier curves and number of cases at risk for the high (scores 1–3) and low (score 0) intra‐tumoral/peri‐tumoral TLS groups of the discovery cohort HSHZ (*n* = 342). The Kaplan–Meier curves and number of cases at risk for the high (scores 1–3) and low (score 0) intra‐tumoral/peri‐tumoral TLS groups of the two validation cohorts Chaoyang C,D) (*n* = 119) and Youan E,F) (*n* = 205). The *p*‐values were calculated by log‐rank test.

To investigate the prognostic value of TLS in intra‐tumoral and peri‐tumoral regions, we built a univariate Cox model to explore the association between intra‐tumoral and peri‐tumoral TLS abundance and recurrence‐free survival (RFS) or overall survival (OS). Specifically, we first divided the samples into TLS‐high (TLS score = 1, 2, or 3) and TLS‐low groups (TLS score = 0) based on their TLS scores. For HSHZ cohort, patients of high intra‐tumoral TLS group had a prolonged RFS and OS (**Figure** **2**A, log‐rank test, *P* < 0.01), while a rather weak correlation was observed between high peri‐tumoral TLS abundance and an improved OS (Figure 2B). Similarly, intra‐tumoral TLS abundance was also positively correlated with both RFS and OS in both Chaoyang and Youan cohorts, the correlation between the peri‐tumoral TLS abundance and RFS or OS was consistently significant in the two cohorts (Figure 2C–E), while a rather weak correlation was observed between peri‐tumoral TLS abundance and OS in Youan cohort (Figure 2B). These results indicated that intra‐tumoral TLS abundance had a higher prognostic value than peri‐tumoral TLS abundance and might serve as a possible prognostic factor in HCC‐LT.

### 2.3 Intra‐Tumoral TLS Abundance is an Independent Prognostic Factor in HCC‐LT

The intra‐tumoral TLS score was closely correlated with RFS and OS in HCC, we investigated whether it was a prognostic factor independent of clinical prognostic indicators. Specifically, AFP level, Milan criteria, PVTT, MVI, maximal tumor diameter, and American Joint Committee on Cancer (AJCC) stage were correlated with RFS or OS in all the three HCC‐LT cohorts by univariate Cox regression analysis (Table S1, Supporting Information, log‐rank test, *P* < 0.05). The prognostic values of these clinical factors were consistent for the HCC‐LT cohorts, which indicated a high quality of the obtained clinical data.

**Figure 3:**
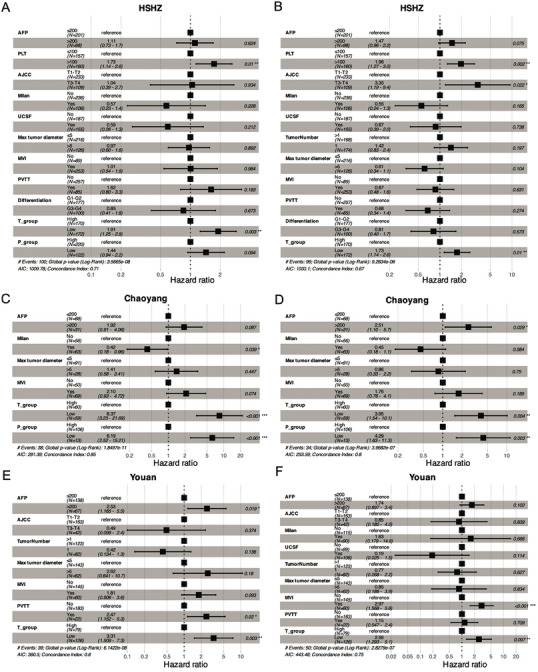
The forest plots for clinicopathological factors in RFS‐ and OS‐based multivariate Cox models. A,B) The hazard ratios, as well as the 95% confidence intervals and statistical significances, in RFS‐ and OS‐based multivariate Cox models for the discovery cohort HSHZ (*n* = 342). C–F) The hazard ratios, as well as the 95% confidence intervals and statistical significances, for the validation cohort Chaoyang (*n* = 119) and Youan (*n* = 205). RFS: recurrence‐free survival, OS: overall survival.

Taking those prognostic variables and TLS scores significantly correlated with RFS or OS, we built RFS‐ and OS‐based multivariate Cox models for each cohort. In HSHZ cohort, intra‐tumoral TLS abundance and PLT were statistically significant in both RFS and OS‐based multivariate Cox models (**Figure** **3**A,B, log‐rank test, *P* < 0.05). Moreover, the intra‐tumoral and peri‐tumoral TLS abundance levels were the significant variables in both RFS‐ and OS‐based Cox models for Chaoyang cohort (Figure 3C,D, log‐rank test, *P* < 0.05). Furthermore, the intra‐tumoral TLS abundance was the only significant variable in both RFS‐ and OS‐based Cox models for the Youan cohort (Figure 3E,F, log‐rank test, *P* < 0.05). These results indicated that intra‐tumoral TLS abundance could be an independent prognostic factor in HCC‐LT.

### 2.4 Clinical Relevance of Intra‐Tumoral TLS Abundance in HCC

**Figure 4:**
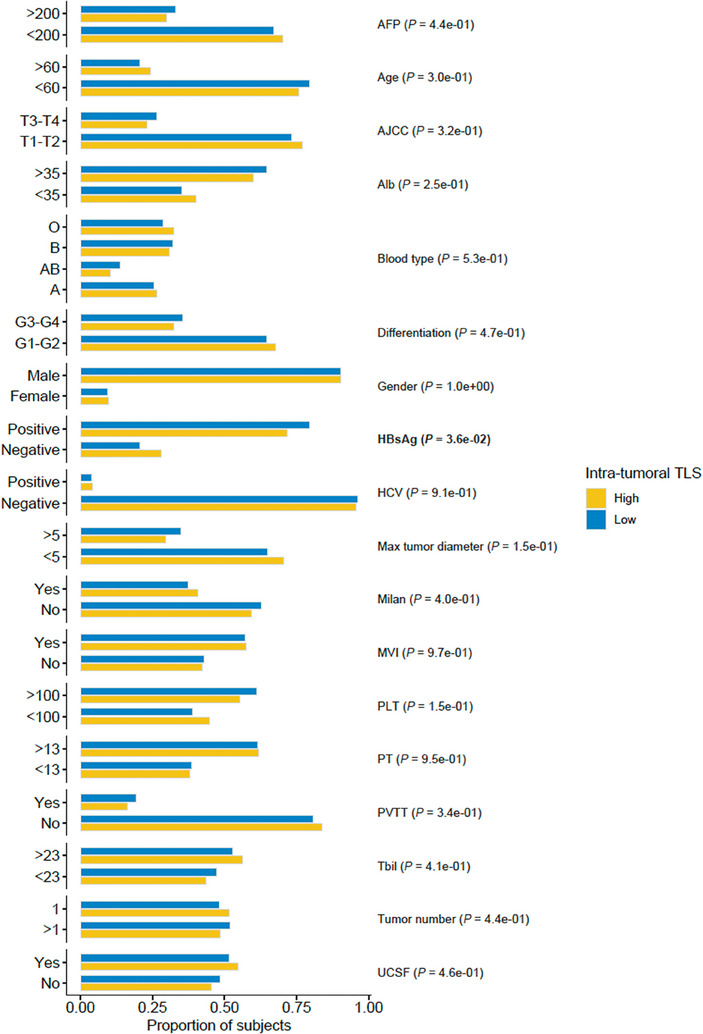
The associations of TLS group with clinicopathological factors in HCC‐LT. The bars represent the percentages of subjects in high (yellow) and low (blue) intra‐tumoral TLS groups (*n* = 666). The association was assessed by Chi‐square test.

Intra‐tumoral TLS abundance could serve as an independent prognostic factor in HCC‐LT, and we investigated its association with 18 clinical or pathological factors in HCC‐LT. Notably, positive HBsAg was significantly associated with intra‐tumoral TLS abundance, and the rate of positive HBsAg was higher in the tumors with low TLS (**Figure** **4**, Chi‐square test, *P* < 0.05). However, some prognosis‐related factors such as AFP, AJCC, max tumor diameter, MVI, PVTT, tumor number, and Milan and UCSF criteria were insignificant, further suggesting that intra‐tumoral TLS was independent of those factors.

### 2.5 Transcriptomic Characterization of HCC Tissues with High and Low TLS Abundance

**Figure 5:**
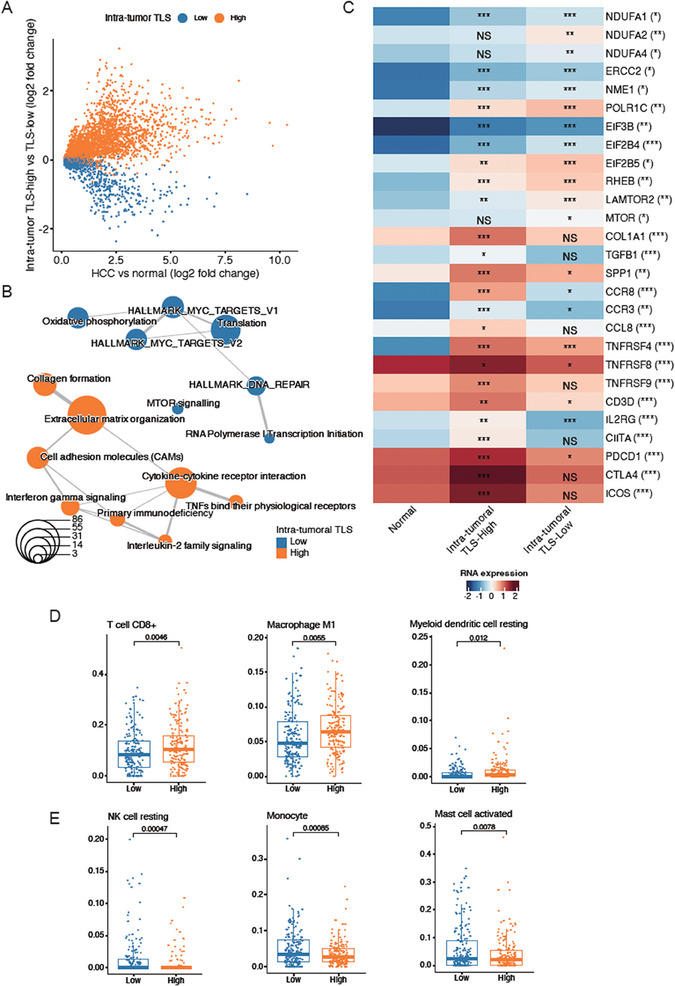
Transcriptomic characteristics of HCC tissues with high and low intra‐tumoral TLS. A) The differentially expressed genes in TLS‐high (orange, *n* = 179) and TLS‐low (blue, *n* = 180) groups. B) The pathways enriched by the differentially expressed genes in TLS‐high and TLS‐low groups. Fisher's exact test assessed the significance. The orange and blue circles represent the pathways for TLS‐high and TLS‐low groups, respectively. C) The representative genes of intra‐tumoral TLS‐high and TLS‐low groups. The immune cells highly infiltrated in the intra‐tumoral D) TLS‐high and E) TLS‐low. The statistical significance was calculated by Wilcoxon rank sum test.

To characterize the transcriptomic signatures in HCC tissues displaying high and low TLS abundance (TLS‐high and TLS‐low groups), we analyzed the gene expression profiles between high intra‐tumoral TLS and low intra‐tumoral TLS groups in TCGA cohort. Interestingly, we identified 2543 signature genes in TLS‐high group and 1215 in TLS‐low group (**Figure** **5**A, adjusted *P* < 0.25 and *P* < 0.05). Consistently, the upregulated genes in intra‐tumoral TLS‐high group (COL1A1, TGFB1, SPP1, CCR8, CCR3, CCL8, TNFRSF4, TNFRSF8, TNFRSF9, CD3D, IL2RG, CIITA, PDCD1, CTLA4, and ICOS) were highly enriched in tumor microenvironment‐related pathways (Figure 5B,C, adjusted *P* < 0.05). In contrast, genes upregulated in intra‐tumoral TLS‐low group were enriched in several hallmarks of cancer, such as protein translation (EIF3B, EIF2B4, EIF2B5), DNA repair (ERCC2, NME1, and POLR1C), oxidative phosphorylation (NDUFA1, NDUFA2, and NDUFA4), and mTOR signaling (RHEB, LAMTOR2, and MTOR) (Figure 5B,C, adjusted *P* < 0.05). These results suggested that the biological characteristics of HCC tumor cells were closely associated with reduced TLS abundance.

As TLS abundance was related with immune cell infiltration, we evaluated and compared immune infiltrations in patients from intra‐tumoral TLS‐high and TLS‐low groups. Consistently, enhanced infiltrations of immune cells, including CD8^+^ T cells, M1 macrophage, and resting dendritic cells (DCs), were observed in TLS‐high group (Figure 5D). Of note, CD8^+^ T cells and M1 macrophage were pro‐inflammatory immune cells, which played key roles in anti‐tumor effect. In contrast with TLS‐high group, higher proportions of resting natural killer (NK) cells, monocytes, activated mast cells were observed in TLS‐low group (Figure 5E). These results suggested that increased infiltrations of those immune cells involved in anti‐tumor activities might contribute to favorable prognoses in TLS‐high group.

**Figure 6:**
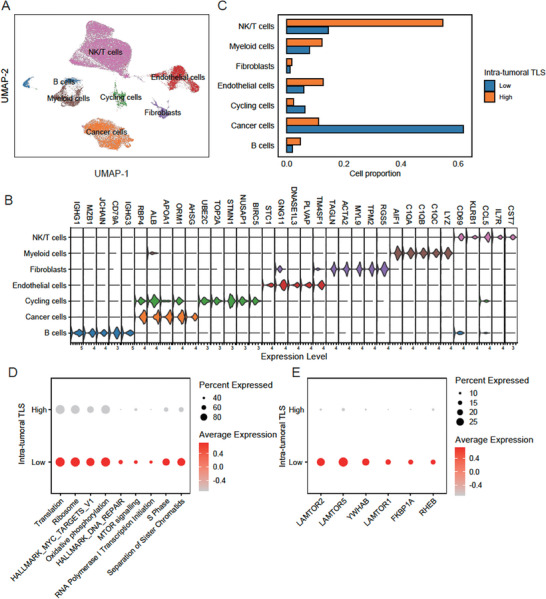
The biological characteristics of cancer cells of intra‐tumoral TLS‐low group by scRNA‐seq data analysis. A) The UMAP dimensional reduction of the single‐cell gene expression profiles. B) The gene expression levels of top‐5 marker genes in the seven cell types. C) The cell proportions of the seven cell types between TLS‐high (*n* = 179) and TLS‐low (*n* = 180) groups. D,E) The module scores for the cancer hallmark‐related pathways and expression of regulators involved in mTOR signaling between TLS‐high and TLS‐low groups. The node size and color represent the percentage of cells expressed and average expression levels, respectively.

As the hallmarks of cancer cells characterized the intra‐tumoral TLS‐low group, we then verified the biological characteristics of this group by a public single‐cell RNA‐seq data^[^ ^14^ ^]^ (GEO accession: GSE156625). Specifically, a total of 57254 cells from 14 HCC tumor samples were clustered into seven cell types (**Figure** **6**A), including cancer cells, fibroblasts, endothelial cells, cycling cells, natural killer (NK), T cells, myeloid cells, and B cells, which were characterized by the marker genes (Figure 6B). By excluding the samples with percentage of cancer cells <1%, we successfully predicted four tumor samples (P4, P7, P8, and P14) as intra‐tumoral TLS‐low cluster, and another 3 (P5, P9, and P13) as intra‐tumoral TLS‐high cluster by Nearest Template Prediction (NTP) algorithm (FDR < 0.25 and *P* < 0.05) (Table S2, Supporting Information). The analysis of the cell type proportions revealed that intra‐tumoral TLS‐low group had a higher proportion of cancer cells, while the intra‐tumoral TLS‐high group had a higher proportion of immune cells, such as B cells, NK/T cells, and myeloid cells (Figure 6C). The module scores for the pathways that upregulated in intra‐tumoral TLS‐low group by bulk RNA‐seq analysis were also upregulated in the cancer cells of that group by scRNA‐seq data (Figure 6D). Particularly, the key regulators of mTOR signaling such as LAMTOR2, LAMTOR5, YWHAB, LAMTOR1, FKBP1A, and RHEB were also upregulated in the cancer cells of intra‐tumoral TLS‐low group. These results further demonstrated that the cancer hallmark‐related pathways, especially mTOR signaling, were significantly activated in cancer cells with low TLS abundance.

### 2.6 Genomic Alterations are Associated with Intra‐Tumoral TLS Abundance

**Figure 7:**
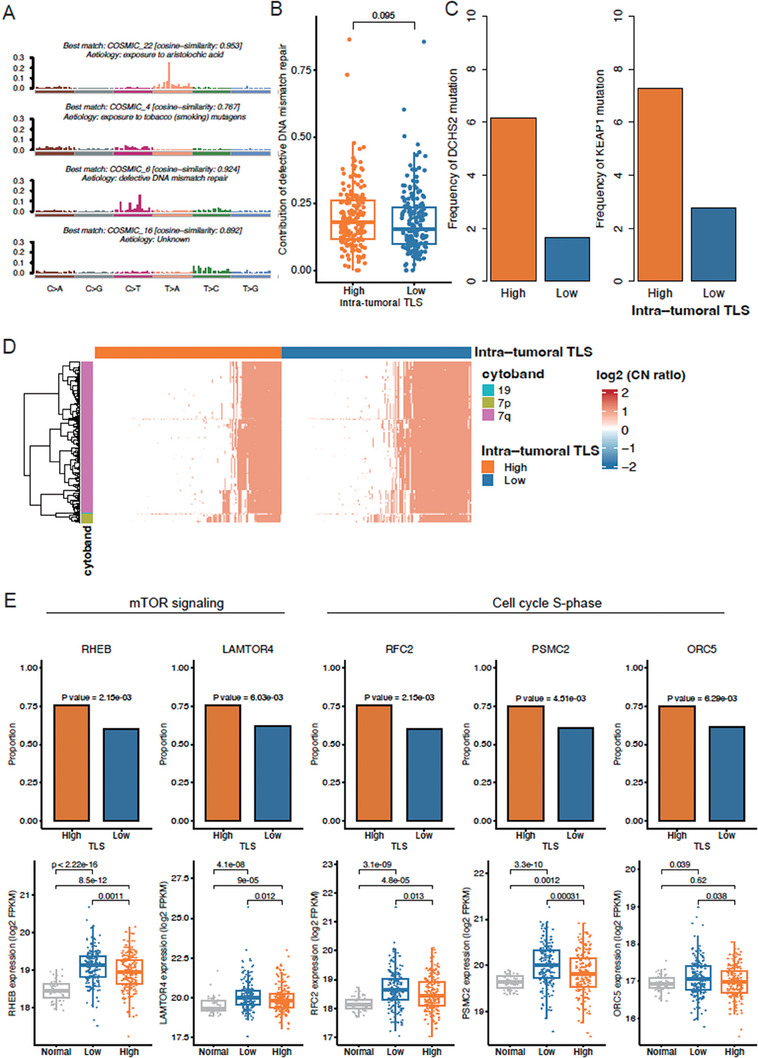
Genomic characteristics of HCC tissues with high and low intra‐tumoral TLS. A) The etiologies identified by the mutational signatures in TCGA HCC. B) The differential contribution of defective DNA mismatch repair between TLS‐high (*n* = 179) and TLS‐low (*n* = 180) groups. The *p*‐value was calculated by Wilcoxon rank sum test. C) The differential frequencies of *DCHS2* and *KEAP1* mutations between TLS‐high and TLS‐low groups. The *p*‐value was calculated by Chi‐square test. D) The genes preferentially amplified or deleted in TLS‐high or TLS‐low group. The color bands on the left represent the cytobands that the genes located within. The red and blue colors in the heatmap represent highly amplified and deleted, respectively, and the white represents the copy number neutral. The color bands on the top represent the TLS‐high and TLS‐low groups. E) The differential frequency and expression levels of amplified genes between normal and TLS‐high and TLS‐low HCC‐LT groups. The significances of differential frequency and expression levels were assessed by Chi‐square test and Wilcoxon rank sum test, respectively.

As the intra‐tumoral TLS‐related clusters showed significantly different gene expression patterns, we then investigated whether the two clusters had distinct genomic signatures. Upon inspecting somatic mutations, we successfully identified four types of mutational signatures, including exposure to aristolochic acid, exposure to tobacco (smoking) mutagens, defective DNA mismatch repair, and unknown etiology, in TCGA cohort (**Figure** **7**A). Specifically, a relatively higher proportion of mutations attributed to defective DNA mismatch repair were observed in intra‐tumoral TLS‐high group than TLS‐low group (Figure 7B, Wilcoxon test, *P* = 0.095), suggesting that high TLS abundance might result from defective DNA mismatch repair in cancer cells. Notably, higher frequencies of *DCHS2* and *KEAP1* mutations were found in intra‐tumoral TLS‐high group than TLS‐low group (Figure 7C, proportion test, *P* < 0.05), suggesting that two genes might promote the tumorigenesis in TLS‐low group.

Furthermore, we also tested whether the copy number alterations (CNAs) in cancer cells were frequently observed in any of the TLS‐related clusters. Surprisingly, the amplifications, especially those located within 7q, were more commonly observed in intra‐tumoral TLS‐low group (Figure 7D, Fisher's exact test, adjusted *P* < 0.05). The regulators involved in mTOR signaling (RHEB and LAMTOR4) and S‐phase (RFC2, PSMC2, and ORC5) were more frequently amplified/gained and highly expressed in intra‐tumoral TLS‐low group (Figure 7E). These results indicated that events like chromosomal instability, mTOR signaling activation, and cell cycle progression occurred more frequently in TLS‐low HCC tissues.

**Figure 8:**
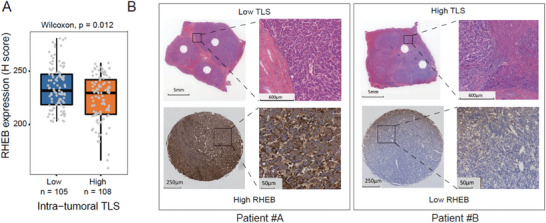
RHEB protein expression in HCC‐LT. A) The differential RHEB protein expression levels between HCC‐LT tissues with high (*n* = 108) and low (*n* = 105) intra‐tumoral TLS abundance by tissue microarray assays (TMA). The *p*‐value was calculated by Wilcoxon rank sum test. B) The RHEB expression in the two representative patients with low (patient #A) and high (patient #B) intra‐tumoral TLS abundance.

To further verify the anti‐correlation between mTOR signaling activation and TLS abundance, we measured the protein expression of RHEB, which was frequently amplified in TLS‐low HCC tissues and acted as a key regulator of mTOR signaling activation. We observed that RHEB was upregulated in TLS‐low HCC tissues by tissue microarray assay (TMA) (**Figure** **8**, Wilcoxon test, *P* < 0.05). These results further indicated that mTOR signaling activation was closely associated with low TLS abundance in HCC tissues.

### 2.7 The Radiomic Model can Accurately Detect Intra‐Tumoral TLS Abundance

**Figure 9:**
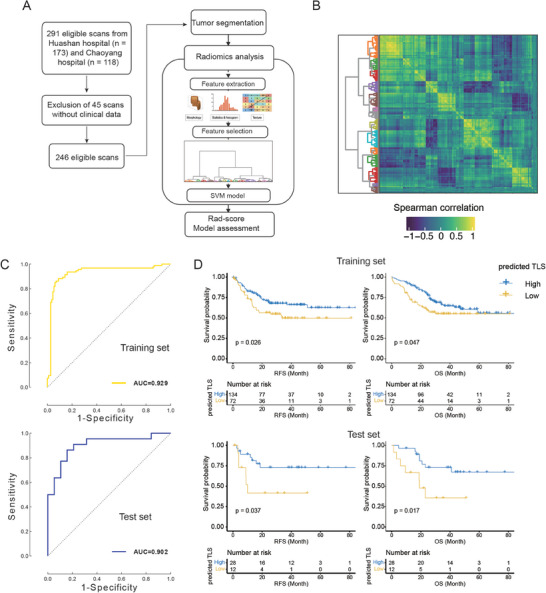
The radiomics‐based model for TLS abundance prediction. A) The workflow for the construction of radiomics‐based model (*n* = 246). B) The spearman correlation between the features was calculated from the radiomic data. C) The model performance in the training (top) and validation (bottom) sets was assessed by the ROC curve/AUC value. D) The Kaplan–Meier curves and the number of cases at risk for the high and intra‐tumoral TLS groups predicted by the radiomic model (top: the training set, bottom: the test set).

To achieve non‐invasive detection of intra‐tumoral TLS abundance in HCC patients, we built a classifier based on radiomics data to distinguish high TLS samples from low TLS ones. After excluding 45 scans from 291 recipients’ scans from Huashan and Chaoyang cohorts, we retained 246 eligible scans for downstream analysis (**Figure** **9**A). Based on Spearman's correlation matrix, 1674 radiomic features were extracted from arterial‐ and venous‐phase CT images for each patient (Figure 9B). Subsequently, the 246 cases were randomly divided into training (n = 206) and test sets (n = 40) (See Experimental Section), and the final radiomics model was built based on the training set using the RBF‐SVM algorithm. After applying this model to both datasets, AUC values of 0.929 and 0.902 were observed in the training and test sets (Figure 9C), respectively. Furthermore, we also found that the subjects with predicted low intra‐tumoral TLS abundance had a worse prognosis than those with predicted high intra‐tumoral TLS abundance (Figure 9D, Log‐rank test, *P* < 0.05). These results suggested that radiomic model could accurately detect intra‐tumoral TLS status and might serve as a promising approach for the detecting of intra‐tumoral TLS abundance in clinical application.

## 3 Discussion

The prognosis of HCC‐LT is affected by tumor recurrence and metastasis. The main contributing factors include tumor heterogeneity, tumor load, and morphological characteristics. The tumor immune microenvironment is an important source of tumor heterogeneity due to its complex components and function.^[^ ^15^ ^]^ Immune cells in the tumor immune microenvironment have a significant impact on the biological behavior of tumors, including tumor occurrence, progression, drug response, and recurrence and metastasis.^[^ ^16^ ^]^

TLS characterized by immune cell aggregates within nonlymphoid tissues, have been recurrently associated with favorable prognoses in HCC. Nevertheless, the potential significance of TLS in the context of HCC after liver transplantation remains largely unexplored. In the course of our investigation, we discerned an absence of correlation between TLS abundance within the intra‐tumoral and peri‐tumoral regions.

Upon deeper inquiry, our study posits that the genesis of TLS within intra‐tumoral domains may be intricately governed by a confluence of factors, including tumor cells, immune cells, and stromal cells. Moreover, our findings suggest that the interplay and communication among these cellular entities within the intricate tapestry of the tumor microenvironment orchestrate the formation of TLS. This aligns with existing literature, which consistently underscores the contributory roles of intra‐tumoral immune cells and stromal cells in the initiation and sustenance of TLS. The nuanced interplay between these cellular components sheds light on the regulatory dynamics underpinning TLS formation and underscores the complexity of immune responses within the tumor milieu.^[^ ^17^, ^18^, ^19^ ^]^

The association between intra‐tumoral TLS abundance and patients’ prognosis and their response to anti‐tumor treatment in resected HCC has been demonstrated.^[^ ^20^, ^21^, ^22^ ^]^ By contrast, we found that the utilization of intra‐tumoral TLS abundance in the prognostic prediction of HCC‐LT may help improve model performance for the first time. Specifically, in univariate Cox models, intra‐tumoral TLS abundance was significantly correlated with both RFS and OS in three HCC‐LT cohorts. Moreover, it was the intra‐tumoral TLS abundance, not the peri‐tumoral one, that was statistically significant in the multivariate Cox models, which employed intra‐tumoral and peri‐tumoral TLS abundance as variables and prognostically relevant clinical factors as co‐factors. These results indicated that intra‐tumoral TLS might serve as a potential prognostic factor in HCC‐LT. Considering that the diseased livers have been completely resected from HCC‐LT patients and the residual tumor cells survived in the form of minimal residual diseases (MRDs) or circulating tumor cells (CTCs), we speculated that the highly specific correlation between intra‐tumoral TLS abundance and RFS/OS and the weak correlation between peri‐tumoral TLS abundance and RFS might be related to the intrinsic biological characteristics of tumor cells and their cellular interactions with immune cells.

In general, the TLS in tumors is accompanied by high infiltrating levels of immune cells, as shown in human lung cancer and breast cancer.^[^ ^23^, ^24^ ^]^ Consistently, we found that tumor tissues in the TLS‐high group of TCGA cohort, in which a higher TLS abundance was detected, were characterized by inflammatory signatures and high infiltration of immune cells. In contrast, the impact of tumor cells on TLS formation remains comparatively little known. For instance, FOXP1 expression in breast cancer cells negatively regulates tumor‐infiltrating lymphocyte migration by inhibiting lymphoid chemokine expression.^[^ ^25^ ^]^ Accordingly, enhanced cancer‐cell‐intrinsic characteristics, such as protein translation (EIF3B, EIF2B4, EIF2B5), DNA repair (ERCC2, NME1, and POLR1C), oxidative phosphorylation (NDUFA1, NDUFA2, and NDUFA4), and mTOR signaling (RHEB, LAMTOR2, and MTOR) were observed in TLS‐low group. By this finding, the regulators involved in mTOR signaling (RHEB and LAMTOR4) and S‐phase (RFC2, PSMC2, and ORC5) were more frequently gained and highly expressed in TLS‐low group, indicating that mTOR signaling activation and cell cycle progression in tumor cells might result in reduced TLS abundance in HCC tissues and unfavorable prognosis in HCC‐LT. Of note, genes involved in mTOR signaling, such as TSC1 and TSC2, were frequently mutated in HCC,^[^ ^26^, ^27^ ^]^ and mTOR signaling was considered as one of the potential therapeutic targets for HCC‐LT with the absence of TLS.^[^ ^28^, ^29^ ^]^ Previous studies have shown that mTOR inhibitors is effective in improving survival, as well as reducing recurrence for HCC patients following LT,^[^ ^30^, ^31^ ^]^ suggesting that HCC‐LT with the absence of TLS might be a potential benefit group of mTOR inhibitors.

Another key feature of this study is that we studied the largest cohort of HCC patients with available radiomics data and built a classifier to detect TLS abundance in a non‐invasive manner. Surprisingly, the classifier achieved a higher performance in detecting intra‐tumoral TLS abundance in both training and test sets, compared with an earlier CT‐based model.^[^ ^13^ ^]^

However, considering the large proportions of HBV‐infected patients, our study is exclusively valuable for eastern HCC‐LT patients, which may be different from Western countries. And over 90% of transplant male patients is another factor, recently sexual disparity is more considered both in liver cirrhosis and transplant prognosis.^[^ ^32^ ^]^ Should the sample size further increase, we envisage that the classifier will become a promising approach for TLS detecting in clinical applications, thereby assisting prognostic prediction and therapeutic strategies for HCC‐LT.

## 4 Conclusion

In summary, reduced intra‐tumoral TLS abundance is associated with enhanced mTOR signaling activation and uncontrolled cell cycle progression in tumor cells, and can serve as an indicator of unfavorable prognosis in HCC‐LT.

## 5 Experimental Section

### Patients and Postoperative Follow‐Up

From January 2016 to December 2021, 666 patients (302 patients from Huashan Hospital, Fudan University, 119 patients from Beijing Chaoyang Hospital affiliated to Capital Medical University, 40 patients from Shulan (Hangzhou) Hospital, Zhejiang Shuren University School of Medicine, and 205 patients from Beijing Youan Hospital, Capital Medical University) who underwent DDLT were enrolled into this study. All transplanted livers were matched by the China Organ Transplant Response System (COTRS) and obtained through Organ Procurement Organizations (OPO) from cardiac or brain death donors. The study protocol was designed and written informed consents were obtained in accordance with the Helsinki Declaration and approved by the ethical committee of the Huashan Hospital. The patients were enrolled based on the inclusion criterion: hepatocellular carcinoma diagnosed by postoperative pathological examination. The exclusion criteria: 1) The presence of extrahepatic tumor metastasis; 2) Death within three months after liver transplantation; 3) Incomplete clinical data. The clinical and pathological information of the enrolled patients, such as laboratory examination data, tumor pathology, postoperative long‐term drug use, including immunosuppressants and targeted drugs, and TNM staging, was collected from the medical records. The follow‐up was performed regularly after discharge from the hospital. A postoperative follow‐up review of blood routine, AFP, PIVKA‐II, liver function and kidney function, blood glucose level, blood lipids level, and blood immunosuppressant concentration was performed. The ultrasound examination was performed every month. Lung Computed Tomography and liver‐enhanced MRI were performed every three months during the first three years, and once half a year three years afterward. A bone scan of Emission Computed Tomography or a PET‐CT was conducted to confirm the recurrence and metastasis if necessary.

### Treatment before the Transplantation

Patients with long expected waiting times for transplantation and tumors beyond Milan criteria were recommended to receive interventional therapy such as TACE, PEI, and RFA to control tumor progression. At the same time, targeted therapy such as sorafenib and lenvatinib and ICIs, were individually or jointly administered as systemic treatment. Supportive treatment was administered to patients with decompensated liver function. Patients who met the Milan criteria were subjected to routine antiviral and supportive treatment.

### Immunosuppressants

All patients were treated with a conventional triple regimen including tacrolimus, mycophenolate mofetil (MMF), and methylprednisolone. An interleukin 2 antagonist was administered on the day of the operation and the fourth postoperative day. The methylprednisolone was withdrawn one month after LT and MMF was withdrawn within 6 months after LT. Transplant patients beyond the Milan criteria were recommended to take sirolimus instead of tacrolimus as an immunosuppressant one month after transplant. Adjuvant targeted therapy such as sorafenib or lenvatinib were given in patients with high AFP level (>200 ng ml^−1^), beyond Milan criteria or poor differentiation. Immunosuppressant blood concentration was monitored weekly during the first three months and twice a month between 3–6 month and monthly 6 months after transplant.

### Post‐Transplant Antiviral Therapy

HBV DNA‐positive patients were treated with a conventional antiviral DAA (tenofovir or entecavir). Hepatitis B immunoglobulin was administered to hepatitis B antigen‐positive patients to maintain adequate hepatitis B surface antibody concentrations. Patients with positive hepatitis C were treated with standard anti‐hepatitis C therapy of Sofosbuvir and Velpatasvir.

### Characterization and Quantification of TLS

To spatially quantify the TLS abundance, the whole slide images were divided into two subregions: intra‐tumoral and peri‐tumoral regions.^[^ ^33^ ^]^ The TLS scoring for the intra‐tumoral region included four categories: 1) score 0 indicated no TLS detected, (2) score 1 indicated 1 or 2 TLS, (3) score 2 represented the region with 3 TLS, and (4) score 3 indicated 4 or more TLS. Similarly, the TLS abundance in peri‐tumoral region could be graded into four categories: 1) score 0 represented no TLS, 2) score 1 indicated TLS detected in the minority of the regions (<25%), 3) score 1 indicated TLS detected in the majority of the regions (25%−75%), 4) score 3 represented TLS detected in almost the entire region (>75%).^[^ ^34^ ^]^

### Bulk RNA‐Seq Data Analysis

UCSC Xena database was used to download gene expression data from 359 HCC tumor samples and 50 adjacent normal samples from TCGA (https://xena.ucsc.edu/), and the expression levels were logarithmically transformed. R limma package was used to analyze differential gene expression.^[^ ^35^ ^]^ The immune cell proportions were estimated based on the gene expression data by CIBERSORT.^[^ ^36^ ^]^

### Single‐Cell RNA‐Seq Data Analysis

A single‐cell RNA‐seq dataset was downloaded from Gene Expression Omnibus (GEO) with accession GSE156625.^[^ ^14^ ^]^ The unique molecular identifier (UMI) count was preprocessed by Seurat^[^ ^37^ ^]^ with mitochondrial percent <20%, the number of RNA features between 500 and 5000. The samples were merged and batch effect was corrected by R Harmony package.^[^ ^38^ ^]^ The study selected 3000 most variable features for principal component analysis (PCA). For the Uniform Manifold Approximation and Projection (UMAP) dimensional reduction and nearest‐neighbor graph construction, Harmony was used.

### Functional Inference for Gene Sets

To infer functionality of identified gene sets, the well‐annotated gene sets grouped according to signaling pathways from MSigDB were obtained using R msigdbr package.^[^ ^39^ ^]^ The hyper‐geometric test was used to measure the significance of enrichment. This analysis was implemented in R cluster Profiler package^[^ ^40^ ^]^ (enricher function).

### Somatic Mutations and Copy Number Alterations

The somatic mutations and copy number alterations (sCNAs) were obtained from UCSC Xena database (https://xena.ucsc.edu/). A total of 55276 somatic mutations from 346 HCC samples were first annotated using ANNOVAR, and then converted to Mutation Annotation Format (MAF). The analysis of somatic mutations was implemented in R maftools package. 220823 CNV segments from 377 HCC samples were investigated to identify the aberrant CNV regions using GISTIC 2.0^[^ ^41^ ^]^ (confidence level = 0.99, q‐value threshold = 0.25, refgene file: human hg38). Significant differences in CNV frequency between TLS‐high and TLS‐low groups were identified using Chi‐square test (*P* < 0.05).

### Tissue Micro‐Array and Immuno‐Histochemistry

The tissue samples were fixed in formaldehyde, paraffin embedded, and mounted on 3‐aminopropyltrioxysilane‐coated slides. A pressure‐cooking procedure in 0.08% citrate buffer for 20 min was used to obtain representative tumor sections from FFPE specimens. Before using the antibodies on the tissue section, the dilution that rendered optimal sensitivity and specificity was determined by tittering the antibodies against the normal tissues. In order to visualize the staining results, sequential incubations were carried out. The same method was used for the negative controls without adding the primary antibody. Using Image Pro‐Plus software, two pathologists independently evaluated immunohistochemical staining without knowing the characteristics of the patient. Image‐Pro Plus 6.0 was used to judge the positivity of all immunohistochemistry pictures using the same clay‐bank color. The cumulative integrated optical density (IOD) of positive signals was calculated for each picture.

### CT Data Acquisition and Analysis

A total of 246 patients with liver tumors were recruited from 2016 to 2021 at two hospitals in this study (138 patients from Huashan hospital and 108 patients from Chaoyang hospital). For the following machine learning analysis in this study, the patient data was split into a training set and a test set with a ratio of 5:1, using stratified sampling based on sex, age, TLS level, and hospital in R software (Version 3.6.2, https://www.r‐project.org/). The 246 patients were grouped into the training set and the test set with a 5:1 training‐to‐test ratio, by stratified sampling according to sex, age, TLS level, and hospital using R software (version 3.6.2, https://www.r‐project.org/). Thus, a training set consisting of 206 patients and a test set of the other 40 patients were obtained. All patients or their guardians gave their informed consent for the utilization of their anonymized CT images and clinical data for research purposes. All the CT images used in this study were acquired using 256‐slice MDCT scanners (Brilliance iCT, Philips, Netherlands) with 128 detector rows, 30 mA tube current, 100 kV tube voltage, 0.9 mm slice thickness, 0.45 mm slice spacing, 0.4 mm pixel size and matrix size of 512 × 512. The injection rate of contrast agent was set at 4.5 mL s^−1^. Finally, CT images taken in arterial and portal venous phases of each patient were obtained. The CT data analysis flowchart is presented in Figure 9A.

### Tumor Segmentation

The tumor segmentation, which incorporated the regions of interest (ROI) that were manually delineated slice‐by‐slice in both arterial‐phase and venous‐phase liver tumor CT images of each patient, was accomplished by two radiologists blinded to the diagnoses using MITK software (Version 2016.3.0, https://www.mitk.org) (Dr. Bin Hu and Dr. Yan Geng), and then reviewed and modified by a senior radiologist (Dr. Daoying Geng).

### Radiomics Analysis

- Radiomic features were extracted from both arterial‐phase and venous‐phase images. According to guidelines from the Image Biomarker Standardization Initiative (IBSI).^[^ ^42^ ^]^ For images from each phase, a total of 1781 radiomics features, which consist of 14 shape‐based features, 18 first‐order statistics features, 75 texture features and 1674 transformed features from images filtered by Laplacian of Gaussian filters with five sigma levels, wavelet filters with eight decompositions, and square, square root, logarithm, exponential, and local binary pattern filters, were extracted with the PyRadiomics (https://pyradiomics.readthedocs.io/en/latest/index.html#). Texture features employed gray‐level matrixes to represent the spatial heterogeneity of intensities within the tumor ROI, with the bin width of intensity being set to 10. The details of all features are described online (https://pyradiomics.readthedocs.io/en/latest/features.html).
- A correlation‐matrix‐based hierarchical clustering method was employed to reduce the dimensionality of input feature space. Specifically, all 3562 radiomics features extracted from both phases were standardized and Spearman's correlation matrix of these features was calculated. Then, features were clustered according to the correlation matrix, to select representative ones and remove irrelevant and redundant features that would diminish the predictive power in further model‐building process, and to control the number of features remaining to within 1/10 of the number of cases to reduce the risk of model overfitting.
- The selected features were input to the radial basis function‐support vector machine (RBF‐SVM) to build a radiomics model.^[^ ^43^ ^]^ By inputting the values of each set of single‐lesion radiomics features into this model, a radiomics‐based score was calculated that reflected the output of the RBF‐SVM model for each patient, and referred it as rad‐score.
- Patients were labeled as TLS‐high or TLS‐low according to their rad‐scores, and the predictive performance of the proposed radiomics model was assessed using ROC curve analysis. The cut‐off for TLS classification was determined using R cutpointr package^[^ ^44^ ^]^ by maximizing the odds ratio, which was calculated as (true positives/ false positives)/ (false negatives/ true negatives). The corresponding area under ROC curve (AUC), accuracy, sensitivity, and specificity values of the model were evaluated in both training and test datasets using R ROCR package.^[^ ^45^ ^]^

### Statistical Analysis

All graphical and statistical analyses were performed using R 4.1.0. Unpaired Wilcoxon rank‐sum tests were used for two‐sample or pairwise mean comparisons. In order to examine the prognostic significance of TLS abundance, as well as other cofactors, the univariate and multivariate Cox models were applied. In the multivariate Cox models, variables with infinitely estimated coefficients were excluded. Chi‐square test was used to test the association between qualitative variables. *p* < 0.05 was considered statistically significant. (^*^ *p* < 0.05, ^**^ *p* < 0.01, ^***^ *p* < 0.001).

ROC curve analysis was used to describe the discriminative power of the radiomics model. Accuracy was calculated by labeling subjects with high TLS as positive cases. Python packages scikit‐learn (https://scikit‐learn.org/) were used for feature selection and model development and validation (Version 3.8.0, https://www.python.org).

## Conflict of Interest

The authors declare no conflict of interest.

## Author Contributions

Z.W., D.G., Q.H., and Y.B. designed this study. Z.W., D.G., Q.H., Y.B., S.Z., J.L., H.X., Y.G., S.L., B.H., S.J., Z.Y., N.Z., Q.Z., J.Z., Y.T., C.S., R.L., and F.T. contributed to data acquisition. J.L., L.Z., H.X., X.L., W.H., Z.F., and G.L. conducted data analysis. J.L., L.Z., H.X., Y.G., S.L., and X.L. interpret the data. J.L., L.Z., H.X., and X.L. wrote the manuscript. All authors read the manuscript and have approved the submitted version.

## Supporting information

Supporting Information

Supporting Information

Supporting Information

## Data Availability

The data that support the findings of this study are available from the corresponding author upon reasonable request.
