# Supplementary material for: The Absence of Intra‐Tumoral Tertiary Lymphoid Structures is Associated with a Worse Prognosis and mTOR Signaling Activation in Hepatocellular Carcinoma with Liver Transplantation: A Multicenter Retrospective Study
Source: Adv Sci (Weinh). 2024 Mar 18;11(21):2309348. doi: 10.1002/advs.202309348 (PMC11151010; doi:10.1002/advs.202309348)
Supplement: Supplementary file 1 — Supporting Information [file ADVS-11-2309348-s002.pdf]

ADVANCED SCIENCE
Open Access

Supporting Information

for *Adv. Sci.*, DOI 10.1002/advs.202309348

The Absence of Intra-Tumoral Tertiary Lymphoid Structures is Associated with a Worse Prognosis and mTOR Signaling Activation in Hepatocellular Carcinoma with Liver Transplantation: A Multicenter Retrospective Study

Jianhua Li, Li Zhang, Hao Xing, Yan Geng, Shaocheng Lv, Xiao Luo, Weiqiao He, Zhi Fu, Guangming Li, Bin Hu, Shengran Jiang, Zhe Yang, Ningqi Zhu, Quanbao Zhang, Jing Zhao, Yifeng Tao, Conghuan Shen, Ruidong Li, Feng Tang, Shusen Zheng, Yun Bao, Qiang He*, Daoying Geng* and Zhengxin Wang**

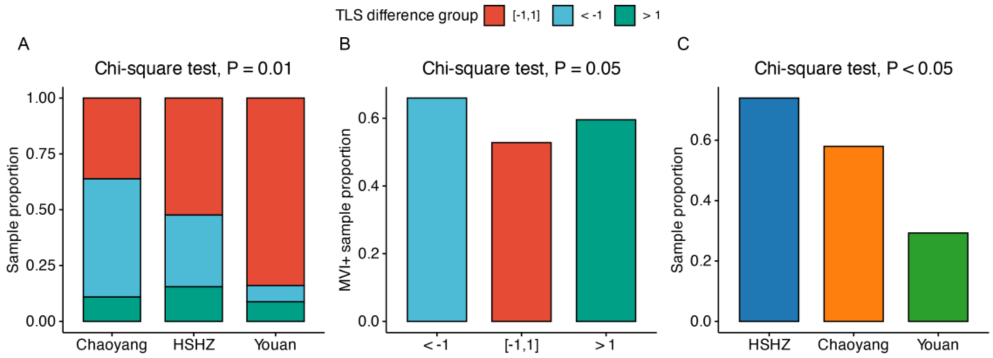

Figure S1. The association of TLS decrease in HCC compared to peri-tumor tissues with MVI presence.

(A) The sample proportion of TLS difference groups in the three HCC-LT cohorts, HSHZ (n = 342), Chaoyang (n = 119), and Youan (n = 205). (B) The proportion of samples with MVI presence in the three TLS difference groups. (C) The proportion of samples with MVI presence in the three HCC-LT cohorts.
